# 4-But­oxy-*N*′-[1-(4-methyl­phen­yl)ethyl­idene]benzohydrazide

**DOI:** 10.1107/S1600536812035921

**Published:** 2012-08-25

**Authors:** Nefise Dilek, Bilal Gunes, Ramazan Gup

**Affiliations:** a Department of Physics, Arts and Sciences Faculty, Aksaray University, 68100 Aksaray, Turkey; b Department of Physics Education, Faculty of Education, Gazi University, Teknikokullar, Ankara, Turkey; c Department of Chemistry, Arts and Sciences Faculty, Mugla University, 48000 Kotekli, Mugla, Turkey

## Abstract

The mol­ecule of the title compound, C_20_H_24_N_2_O_2_, exists in a *trans* conformation with respect to the C=N bond. The dihedral angle between the benzene rings is 79.0 (1)°. In the crystal, N—H⋯O hydrogen bonds link the mol­ecules into chains propagating in [001]. Two weak C—H⋯O inter­actions also occur.

## Related literature

For acyl­hydrazone compounds, see: Rollas & Küçükgüzel (2007 ▶); Vicini *et al.* (2006 ▶); Chimenti *et al.* (2007 ▶). For aroylhydrazone compounds, see: Barbazan *et al.*, 2008 ▶; Dang *et al.*, 2007 ▶. Hydrazones typically act as bi- and tridentate, mono or biprotic depending on the reaction conditions, see: Gup & Kirkan (2005 ▶); Naskar *et al.* (2004 ▶); Sreeja *et al.* (2003 ▶). For bond lengths and angles in similar structures, see: Li & Ban (2009 ▶); Mao *et al.* (2011 ▶); Singh & Singh (2010 ▶).
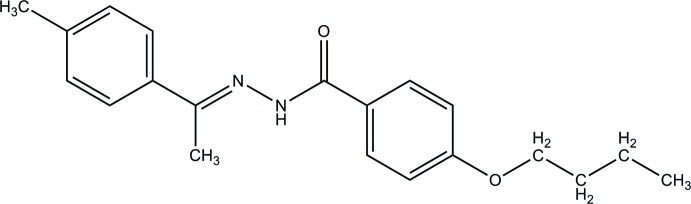


## Experimental

### 

#### Crystal data

C_20_H_24_N_2_O_2_
*M* *_r_* = 324.41
Monoclinic, 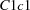

*a* = 15.0800 (4) Å
*b* = 14.0134 (4) Å
*c* = 8.2419 (2) Å
β = 94.609 (2)°
*V* = 1736.06 (8) Å^3^
*Z* = 4
Mo *K*α radiation
μ = 0.08 mm^−1^
*T* = 105 K
0.38 × 0.21 × 0.17 mm

#### Data collection

Bruker APEXII CCD diffractometer
Absorption correction: multi-scan (Blessing, 1995 ▶) *T*_min_ = 0.970, *T*_max_ = 0.987
8163 measured reflections
2150 independent reflections
1980 reflections with *I* > 2σ(*I*)
*R*_int_ = 0.026

#### Refinement

*R*[*F* ^2^ > 2σ(*F* ^2^)] = 0.039
*wR*(*F* ^2^) = 0.101
*S* = 0.93
2150 reflections
220 parameters
2 restraints
H-atom parameters constrained
Δρ_max_ = 0.38 e Å^−3^
Δρ_min_ = −0.20 e Å^−3^

### 

Data collection: *APEX2* (Bruker, 2005 ▶); cell refinement: *SAINT* (Bruker, 2005 ▶); data reduction: *SAINT*; program(s) used to solve structure: *SHELXS97* (Sheldrick, 2008 ▶); program(s) used to refine structure: *SHELXL97* (Sheldrick, 2008 ▶); molecular graphics: *ORTEP* (Farrugia, 1997 ▶) and *PLATON* (Spek, 2009 ▶); software used to prepare material for publication: *WinGX* (Farrugia, 1999 ▶).

## Supplementary Material

Crystal structure: contains datablock(s) I. DOI: 10.1107/S1600536812035921/bq2373sup1.cif

Structure factors: contains datablock(s) I. DOI: 10.1107/S1600536812035921/bq2373Isup2.hkl

Supplementary material file. DOI: 10.1107/S1600536812035921/bq2373Isup3.cml

Additional supplementary materials:  crystallographic information; 3D view; checkCIF report

## Figures and Tables

**Table 1:** Hydrogen-bond geometry (Å, °)

*D*—H⋯*A*	*D*—H	H⋯*A*	*D*⋯*A*	*D*—H⋯*A*
N1—H1⋯O2^i^	0.88	2.15	2.975 (2)	155
C16—H16*C*⋯O2^i^	0.98	2.57	3.307 (3)	131
C17—H17⋯O2^ii^	0.95	2.59	3.527 (3)	168

Symmetry codes: (i) 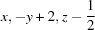; (ii) 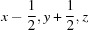.

**Fig. 1.**
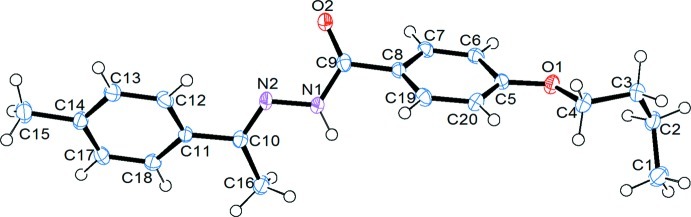
An ORTEP drawing of molecular structure with the crystallographic numbering scheme. Thermal ellipsoids are drawn at 30% probability levels.

**Fig. 2.**
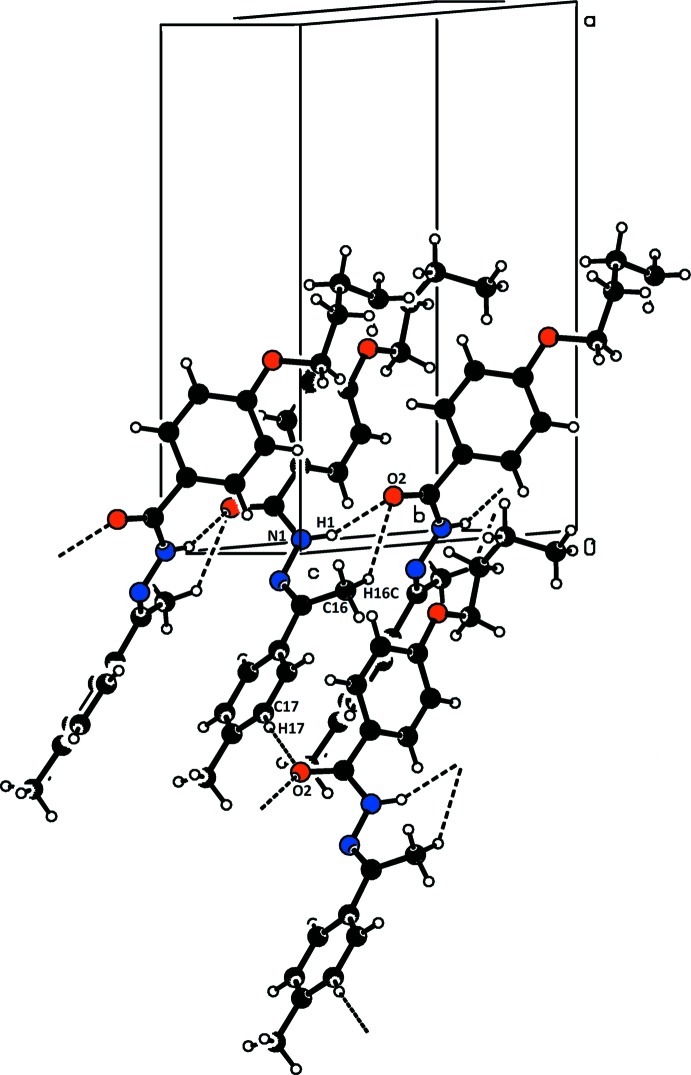
A packing diagram for (I), projected along c direction. Hydrogen bonds are indicated by dashed lines.

## supplementary crystallographic information

### Comment

Acylhydrazones and their derivatives constitute a versatile class of compounds in organic and coordination chemistry. These compounds have interesting biological properties, such as anti-inflammatory, analgesic, anticonvulsant, antituberculous, antitumor, anti-HIV and antimicrobial activity (Rollas & Küçükgüzel, 2007; Vicini *et al.*, 2006; Chimenti *et al.*, 2007).

Aroylhydrazones are important compounds for drug design, as possible ligands for metal complexes, catalysis and also for the syntheses of heterocyclic compounds (Barbazan *et al.*, 2008; Dang *et al.*, 2007). The ease of preparation, increased hydrolytic stability relative to imines, and tendency toward crystallinity are all desirable characteristics of hydrazones. Due to these positive traits, the chemical properties of aroylhydrazones have been extensively studied for a long time. Acylhydrazones possess two connected nitrogen atoms of different nature and a carbon-nitrogen double bond that is conjugated with a lone electron pair of the terminal nitrogen atom. These structural fragments are mainly responsible for the physical and chemical properties of hydrazones. The introduction of functional groups in the hydrazone molecules expands the scope of use in coordination chemistry.

Aroylhydrazones are potential ligands due to having a number of bonding sites. They can act a neutral or monoanionic bidentate or tridentate ligand depending on the substituents and the reaction conditions. Furthermore, abilities to coordinate to metals either in keto (I) or enol (II) tautomeric form make them attractive as ligands. This compound is in the keto form in the solid state. The keto hydrazone moiety may coordinate to metals in the keto amide or deprotonated enolimine form. Hydrazones typically act as bi- and tridentate, mono or biprotic depending on the reaction conditions (Sreeja *et al.*, 2003; Naskar *et al.*, 2004; Gup & Kirkan, 2005).

The crystal structure is shown in Fig. 1 with atom-numbering scheme. The bond lengths and angles are in the normal ranges in the molecule. The molecule exist in a *trans* configuration with respect to the C10═N2 [1.286 (3) Å] bond and the torsion angle N1—N2—C10—C11 = 176.6 (2)°. The O1—C5, O1—C4 and O2═C9 bond lengths are 1.363 (3) Å, 1.444 (3) Å and 1.229 (3) Å, respectively. The N1—C9 and N1—N2 bond lengths are 1.356 (3) Å and 1.390 (3) Å. The other bond lengths and angles in the molecule are within expected ranges, and similar to the other studies (Singh & Singh, 2010; Li & Ban, 2009; Mao *et al.*, 2011).

The ring A (C5–C8, C19, C20) and B (C11–C14, C17, C18) are each essentially planar. The dihedral angle between two substituted benzene rings is 79.0 (1)°, indicating the Schiff base molecule is twisted. The N1 atom lie above 0.937 (4) Å from the A plane. The N2 atom lie above 0.585 (4) Å from the B plane.

As can be seen from the packing diagram (Fig. 2), inter-molecular N—H···O and C—H···O hydrogen bonds (Table 1) link the molecules and these hydrogen bonds may be effective in the stabilization of the crystal structure. In these interactions, there are the N1, C16 and C17 atoms of molecule as donor and the O2 atom of the other molecules as acceptor (Table 1, Fig. 2).

### Experimental

4'-Methylacetophenon (4 mmol, 0.552 g) dissolved in ethanol (10 ml) was added dropwise to a suspension of 4-hydroxybenzohydrazide (4 mmol, 0.608 g) with two drops of glacial acetic acid in ethanol (40 ml) in room temperature. The reaction mixture was refluxed for further 8 h and the colorless product was filtered. The pure hydrazone was collected by crystallization from ethanol.

A mixture 4-hydroxy-*N*'-[(1E)-1-(4-methylphenyl)ethylidene]benzohydrazide (10 mmol, 2.68 g), 1-bromobutane (10 mmol, 1.370 g) and dry K_2_CO_3_ (10 mmol, 1.380 g) in 40 ml acetone was refluxed with stirring for 24 h and poured to 200 ml of cold water. The white precipitate formed was filtered and washed with water and finally recrystallized from acetone-water. Chemical structure of title compound is given (I).

### Refinement

The H atoms were positioned geometrically, with C—H = 0.95 Å, N—H = 0.88 Å, and and constrained to ride on their parent atoms, with *U*_iso_(H) = 1.2*U*_eq_(C,N). Also, the methyl H atoms were positioned geometrically, with C—H = 0.98 Å and *U*_iso_(H) = 1.5*U*_eq_(C). The absolute structure could not be determined and 1089 Friedel pairs were averaged before the last refinement.

### Crystal data

**Table:**

C_20_H_24_N_2_O_2_	*F*(000) = 696
*M**_r_* = 324.41	*D*_x_ = 1.241 Mg m^−^^3^
Monoclinic, *C*1*c*1	Mo *K*α radiation, λ = 0.71073 Å
Hall symbol: C-2yc	Cell parameters from 3295 reflections
*a* = 15.0800 (4) Å	θ = 2.7–28.2°
*b* = 14.0134 (4) Å	µ = 0.08 mm^−^^1^
*c* = 8.2419 (2) Å	*T* = 105 K
β = 94.609 (2)°	Prism, colourless
*V* = 1736.06 (8) Å^3^	0.38 × 0.21 × 0.17 mm
*Z* = 4	

### Data collection

**Table:**

Bruker APEXII CCD diffractometer	2150 independent reflections
Radiation source: sealed tube	1980 reflections with *I* > 2σ(*I*)
Graphite monochromator	*R*_int_ = 0.026
φ and ω scans	θ_max_ = 28.4°, θ_min_ = 2.0°
Absorption correction: multi-scan (Blessing, 1995)	*h* = −20→14
*T*_min_ = 0.970, *T*_max_ = 0.987	*k* = −18→17
8163 measured reflections	*l* = −10→10

### Refinement

**Table:**

Refinement on *F*^2^	Primary atom site location: structure-invariant direct methods
Least-squares matrix: full	Secondary atom site location: difference Fourier map
*R*[*F*^2^ > 2σ(*F*^2^)] = 0.039	Hydrogen site location: inferred from neighbouring sites
*wR*(*F*^2^) = 0.101	H-atom parameters constrained
*S* = 0.93	*w* = 1/[σ^2^(*F*_o_^2^) + (0.0677*P*)^2^ + 0.7866*P*] where *P* = (*F*_o_^2^ + 2*F*_c_^2^)/3
2150 reflections	(Δ/σ)_max_ < 0.001
220 parameters	Δρ_max_ = 0.38 e Å^−^^3^
2 restraints	Δρ_min_ = −0.20 e Å^−^^3^

### Special details

**Table:**

Geometry. All e.s.d.'s (except the e.s.d. in the dihedral angle between two l.s. planes) are estimated using the full covariance matrix. The cell e.s.d.'s are taken into account individually in the estimation of e.s.d.'s in distances, angles and torsion angles; correlations between e.s.d.'s in cell parameters are only used when they are defined by crystal symmetry. An approximate (isotropic) treatment of cell e.s.d.'s is used for estimating e.s.d.'s involving l.s. planes.
Refinement. Refinement of *F*^2^ against ALL reflections. The weighted *R*-factor *wR* and goodness of fit *S* are based on *F*^2^, conventional *R*-factors *R* are based on *F*, with *F* set to zero for negative *F*^2^. The threshold expression of *F*^2^ > σ(*F*^2^) is used only for calculating *R*-factors(gt) *etc*. and is not relevant to the choice of reflections for refinement. *R*-factors based on *F*^2^ are statistically about twice as large as those based on *F*, and *R*-factors based on ALL data will be even larger.

### Fractional atomic coordinates and isotropic or equivalent isotropic displacement parameters (Å^2^)

**Table:**

	*x*	*y*	*z*	*U*_iso_*/*U*_eq_	
O1	0.37326 (10)	0.84731 (12)	0.17841 (19)	0.0229 (4)	
O2	0.09570 (11)	0.91173 (11)	0.70734 (19)	0.0221 (3)	
N1	0.02616 (12)	0.99195 (14)	0.4926 (2)	0.0206 (4)	
H1	0.0300	1.0165	0.3952	0.025*	
N2	−0.04815 (12)	1.00759 (14)	0.5785 (2)	0.0204 (4)	
C1	0.47160 (18)	0.8908 (2)	−0.2273 (3)	0.0316 (6)	
H1A	0.4698	0.8420	−0.3127	0.047*	
H1B	0.5106	0.9431	−0.2557	0.047*	
H1C	0.4114	0.9154	−0.2174	0.047*	
C2	0.50729 (16)	0.84699 (19)	−0.0661 (3)	0.0265 (5)	
H2A	0.5691	0.8250	−0.0755	0.032*	
H2B	0.5089	0.8966	0.0195	0.032*	
C3	0.45168 (16)	0.76347 (18)	−0.0149 (3)	0.0261 (5)	
H3A	0.4450	0.7172	−0.1059	0.031*	
H3B	0.4843	0.7310	0.0785	0.031*	
C4	0.36010 (16)	0.79010 (17)	0.0327 (3)	0.0236 (5)	
H4A	0.3274	0.8269	−0.0555	0.028*	
H4B	0.3255	0.7320	0.0540	0.028*	
C5	0.30078 (15)	0.86690 (15)	0.2613 (3)	0.0186 (4)	
C6	0.31990 (15)	0.90727 (16)	0.4156 (3)	0.0198 (4)	
H6	0.3799	0.9204	0.4533	0.024*	
C7	0.25217 (14)	0.92824 (15)	0.5135 (3)	0.0192 (4)	
H7	0.2658	0.9530	0.6199	0.023*	
C8	0.16373 (14)	0.91299 (15)	0.4559 (3)	0.0181 (4)	
C9	0.09251 (14)	0.93751 (15)	0.5645 (3)	0.0191 (4)	
C10	−0.09609 (14)	1.08078 (16)	0.5367 (3)	0.0190 (4)	
C11	−0.17777 (14)	1.09221 (16)	0.6243 (3)	0.0179 (4)	
C12	−0.21596 (15)	1.01299 (16)	0.6959 (3)	0.0217 (5)	
H12	−0.1916	0.9512	0.6826	0.026*	
C13	−0.28925 (15)	1.02417 (17)	0.7862 (3)	0.0230 (5)	
H13	−0.3136	0.9700	0.8358	0.028*	
C14	−0.32750 (15)	1.11314 (17)	0.8052 (3)	0.0218 (5)	
C15	−0.40582 (17)	1.1251 (2)	0.9068 (3)	0.0296 (5)	
H15A	−0.4605	1.1320	0.8350	0.044*	
H15B	−0.3971	1.1822	0.9749	0.044*	
H15C	−0.4107	1.0690	0.9764	0.044*	
C16	−0.07349 (17)	1.15369 (17)	0.4137 (3)	0.0257 (5)	
H16A	−0.0238	1.1930	0.4591	0.039*	
H16B	−0.1254	1.1944	0.3861	0.039*	
H16C	−0.0565	1.1214	0.3154	0.039*	
C17	−0.29159 (15)	1.19105 (16)	0.7300 (3)	0.0224 (5)	
H17	−0.3177	1.2523	0.7398	0.027*	
C18	−0.21795 (15)	1.18082 (16)	0.6403 (3)	0.0212 (5)	
H18	−0.1947	1.2351	0.5893	0.025*	
C19	0.14483 (15)	0.87441 (15)	0.3012 (3)	0.0211 (4)	
H19	0.0847	0.8651	0.2608	0.025*	
C20	0.21321 (15)	0.84930 (16)	0.2050 (3)	0.0210 (5)	
H20	0.1999	0.8204	0.1017	0.025*	

### Atomic displacement parameters (Å^2^)

**Table:**

	*U*^11^	*U*^22^	*U*^33^	*U*^12^	*U*^13^	*U*^23^
O1	0.0193 (8)	0.0298 (8)	0.0200 (8)	0.0002 (7)	0.0046 (6)	−0.0030 (7)
O2	0.0206 (8)	0.0230 (8)	0.0234 (8)	0.0035 (6)	0.0065 (6)	0.0007 (7)
N1	0.0184 (9)	0.0230 (10)	0.0213 (9)	0.0036 (7)	0.0079 (7)	0.0040 (7)
N2	0.0155 (9)	0.0244 (9)	0.0221 (9)	0.0012 (7)	0.0073 (7)	0.0001 (8)
C1	0.0303 (14)	0.0420 (14)	0.0229 (12)	−0.0006 (11)	0.0049 (10)	0.0029 (11)
C2	0.0195 (11)	0.0408 (14)	0.0199 (11)	0.0031 (9)	0.0054 (9)	−0.0002 (10)
C3	0.0264 (12)	0.0311 (12)	0.0214 (10)	0.0085 (9)	0.0061 (9)	−0.0001 (10)
C4	0.0249 (11)	0.0263 (11)	0.0202 (10)	0.0001 (9)	0.0063 (8)	−0.0031 (9)
C5	0.0186 (10)	0.0174 (10)	0.0202 (10)	0.0021 (8)	0.0046 (8)	0.0019 (8)
C6	0.0170 (10)	0.0211 (11)	0.0210 (11)	0.0005 (8)	−0.0011 (8)	0.0020 (8)
C7	0.0200 (11)	0.0159 (10)	0.0215 (11)	0.0030 (8)	0.0012 (9)	−0.0002 (8)
C8	0.0173 (11)	0.0150 (9)	0.0228 (11)	0.0021 (7)	0.0061 (8)	0.0030 (8)
C9	0.0169 (10)	0.0164 (10)	0.0247 (11)	−0.0017 (8)	0.0070 (8)	−0.0005 (8)
C10	0.0187 (10)	0.0179 (10)	0.0209 (10)	−0.0005 (8)	0.0045 (8)	−0.0008 (8)
C11	0.0161 (10)	0.0215 (10)	0.0162 (10)	0.0014 (8)	0.0020 (8)	−0.0004 (9)
C12	0.0207 (11)	0.0180 (11)	0.0263 (11)	0.0029 (8)	0.0021 (8)	0.0023 (9)
C13	0.0186 (10)	0.0249 (11)	0.0259 (11)	0.0003 (9)	0.0033 (9)	0.0044 (9)
C14	0.0171 (10)	0.0317 (12)	0.0167 (10)	0.0012 (9)	0.0025 (8)	−0.0017 (9)
C15	0.0213 (12)	0.0370 (14)	0.0313 (13)	0.0036 (10)	0.0075 (10)	−0.0024 (11)
C16	0.0295 (12)	0.0216 (11)	0.0275 (12)	0.0023 (9)	0.0114 (10)	0.0033 (9)
C17	0.0203 (11)	0.0217 (11)	0.0252 (11)	0.0045 (8)	0.0023 (9)	−0.0032 (9)
C18	0.0232 (11)	0.0188 (10)	0.0218 (11)	0.0006 (8)	0.0031 (9)	0.0014 (9)
C19	0.0152 (10)	0.0211 (10)	0.0270 (11)	−0.0007 (8)	0.0018 (8)	0.0009 (9)
C20	0.0214 (11)	0.0238 (11)	0.0181 (10)	−0.0002 (8)	0.0046 (8)	−0.0009 (9)

### Geometric parameters (Å, º)

**Table:**

O1—C5	1.363 (3)	C8—C19	1.393 (3)
O1—C4	1.444 (3)	C8—C9	1.492 (3)
O2—C9	1.229 (3)	C10—C11	1.485 (3)
N1—C9	1.356 (3)	C10—C16	1.498 (3)
N1—N2	1.390 (2)	C11—C18	1.393 (3)
N1—H1	0.8800	C11—C12	1.403 (3)
N2—C10	1.286 (3)	C12—C13	1.390 (3)
C1—C2	1.522 (3)	C12—H12	0.9500
C1—H1A	0.9800	C13—C14	1.388 (3)
C1—H1B	0.9800	C13—H13	0.9500
C1—H1C	0.9800	C14—C17	1.387 (3)
C2—C3	1.519 (4)	C14—C15	1.511 (3)
C2—H2A	0.9900	C15—H15A	0.9800
C2—H2B	0.9900	C15—H15B	0.9800
C3—C4	1.512 (3)	C15—H15C	0.9800
C3—H3A	0.9900	C16—H16A	0.9800
C3—H3B	0.9900	C16—H16B	0.9800
C4—H4A	0.9900	C16—H16C	0.9800
C4—H4B	0.9900	C17—C18	1.390 (3)
C5—C20	1.386 (3)	C17—H17	0.9500
C5—C6	1.400 (3)	C18—H18	0.9500
C6—C7	1.383 (3)	C19—C20	1.396 (3)
C6—H6	0.9500	C19—H19	0.9500
C7—C8	1.396 (3)	C20—H20	0.9500
C7—H7	0.9500		
			
C5—O1—C4	117.82 (17)	O2—C9—C8	122.15 (19)
C9—N1—N2	117.58 (17)	N1—C9—C8	114.14 (19)
C9—N1—H1	121.2	N2—C10—C11	115.23 (19)
N2—N1—H1	121.2	N2—C10—C16	124.9 (2)
C10—N2—N1	116.62 (18)	C11—C10—C16	119.86 (19)
C2—C1—H1A	109.5	C18—C11—C12	118.0 (2)
C2—C1—H1B	109.5	C18—C11—C10	121.8 (2)
H1A—C1—H1B	109.5	C12—C11—C10	120.24 (19)
C2—C1—H1C	109.5	C13—C12—C11	120.5 (2)
H1A—C1—H1C	109.5	C13—C12—H12	119.8
H1B—C1—H1C	109.5	C11—C12—H12	119.8
C3—C2—C1	112.9 (2)	C14—C13—C12	121.2 (2)
C3—C2—H2A	109.0	C14—C13—H13	119.4
C1—C2—H2A	109.0	C12—C13—H13	119.4
C3—C2—H2B	109.0	C17—C14—C13	118.4 (2)
C1—C2—H2B	109.0	C17—C14—C15	120.7 (2)
H2A—C2—H2B	107.8	C13—C14—C15	120.9 (2)
C4—C3—C2	114.7 (2)	C14—C15—H15A	109.5
C4—C3—H3A	108.6	C14—C15—H15B	109.5
C2—C3—H3A	108.6	H15A—C15—H15B	109.5
C4—C3—H3B	108.6	C14—C15—H15C	109.5
C2—C3—H3B	108.6	H15A—C15—H15C	109.5
H3A—C3—H3B	107.6	H15B—C15—H15C	109.5
O1—C4—C3	106.59 (19)	C10—C16—H16A	109.5
O1—C4—H4A	110.4	C10—C16—H16B	109.5
C3—C4—H4A	110.4	H16A—C16—H16B	109.5
O1—C4—H4B	110.4	C10—C16—H16C	109.5
C3—C4—H4B	110.4	H16A—C16—H16C	109.5
H4A—C4—H4B	108.6	H16B—C16—H16C	109.5
O1—C5—C20	125.24 (19)	C14—C17—C18	120.9 (2)
O1—C5—C6	114.95 (19)	C14—C17—H17	119.5
C20—C5—C6	119.81 (19)	C18—C17—H17	119.5
C7—C6—C5	120.5 (2)	C17—C18—C11	121.0 (2)
C7—C6—H6	119.7	C17—C18—H18	119.5
C5—C6—H6	119.7	C11—C18—H18	119.5
C6—C7—C8	120.0 (2)	C8—C19—C20	120.8 (2)
C6—C7—H7	120.0	C8—C19—H19	119.6
C8—C7—H7	120.0	C20—C19—H19	119.6
C19—C8—C7	119.36 (19)	C5—C20—C19	119.5 (2)
C19—C8—C9	122.27 (19)	C5—C20—H20	120.3
C7—C8—C9	118.4 (2)	C19—C20—H20	120.3
O2—C9—N1	123.69 (19)		
			
C9—N1—N2—C10	158.6 (2)	N2—C10—C11—C18	155.7 (2)
C1—C2—C3—C4	−68.9 (3)	C16—C10—C11—C18	−22.4 (3)
C5—O1—C4—C3	−169.15 (18)	N2—C10—C11—C12	−23.3 (3)
C2—C3—C4—O1	−65.7 (3)	C16—C10—C11—C12	158.6 (2)
C4—O1—C5—C20	−10.6 (3)	C18—C11—C12—C13	−2.9 (3)
C4—O1—C5—C6	168.89 (18)	C10—C11—C12—C13	176.1 (2)
O1—C5—C6—C7	−178.39 (19)	C11—C12—C13—C14	1.3 (4)
C20—C5—C6—C7	1.1 (3)	C12—C13—C14—C17	0.8 (3)
C5—C6—C7—C8	−2.8 (3)	C12—C13—C14—C15	−178.6 (2)
C6—C7—C8—C19	1.6 (3)	C13—C14—C17—C18	−1.3 (3)
C6—C7—C8—C9	−179.34 (19)	C15—C14—C17—C18	178.1 (2)
N2—N1—C9—O2	−9.6 (3)	C14—C17—C18—C11	−0.4 (3)
N2—N1—C9—C8	171.93 (18)	C12—C11—C18—C17	2.4 (3)
C19—C8—C9—O2	130.8 (2)	C10—C11—C18—C17	−176.6 (2)
C7—C8—C9—O2	−48.2 (3)	C7—C8—C19—C20	1.2 (3)
C19—C8—C9—N1	−50.6 (3)	C9—C8—C19—C20	−177.8 (2)
C7—C8—C9—N1	130.3 (2)	O1—C5—C20—C19	−178.9 (2)
N1—N2—C10—C11	176.57 (18)	C6—C5—C20—C19	1.7 (3)
N1—N2—C10—C16	−5.4 (3)	C8—C19—C20—C5	−2.9 (3)

### Hydrogen-bond geometry (Å, º)

**Table:**

*D*—H···*A*	*D*—H	H···*A*	*D*···*A*	*D*—H···*A*
N1—H1···O2^i^	0.88	2.15	2.975 (2)	155
C16—H16*C*···O2^i^	0.98	2.57	3.307 (3)	131
C17—H17···O2^ii^	0.95	2.59	3.527 (3)	168

Symmetry codes: (i) *x*, −*y*+2, *z*−1/2; (ii) *x*−1/2, *y*+1/2, *z*.
